# The Effects of Reversibility of H2-H3 Phase Transition on Ni-Rich Layered Oxide Cathode for High-Energy Lithium-Ion Batteries

**DOI:** 10.3389/fchem.2019.00500

**Published:** 2019-07-16

**Authors:** Jie Chen, Huiping Yang, Tianhao Li, Chaoyang Liu, Hui Tong, Jiaxin Chen, Zengsheng Liu, Lingfeng Xia, Zhaoyong Chen, Junfei Duan, Lingjun Li

**Affiliations:** ^1^School of Materials Science and Engineering, Changsha University of Science and Technology, Changsha, China; ^2^School of Metallurgy and Environment, Central South University, Changsha, China; ^3^Hunan Provincial Key Laboratory of Modeling and Monitoring on the Near-Earth Electromagnetic Environments, Changsha University of Science and Technology, Changsha, China

**Keywords:** lithium ion batteries, cathode materials, LiNi_0.8_Co_0.1_Mn_0.1_O_2_, Ti-doped, phase transitions

## Abstract

Although LiNi_0.8_Co_0.1_Mn_0.1_O_2_ is attracting increasing attention on account of its high specific capacity, the moderate cycle lifetime still hinders its large-scale commercialization applications. Herein, the Ti-doped LiNi_0.8_Co_0.1_Mn_0.1_O_2_ compounds are successfully synthesized. The Li(Ni_0.8_Co_0.1_Mn_0.1_)_0.99_Ti_0.01_O_2_ sample exhibits the best electrochemical performance. Under the voltage range of 2.7**–**4.3 V, it maintains a reversible capacity of 151.01 mAh·g^−1^ with the capacity retention of 83.98% after 200 cycles at 1 C. Electrochemical impedance spectroscopy (EIS) and differential capacity profiles during prolonged cycling demonstrate that the Ti doping could enhance both the abilities of electronic transition and Li ion diffusion. More importantly, Ti doping can also improve the reversibility of the H2-H3 phase transitions during charge-discharge cycles, thus improving the electrochemical performance of Ni-rich cathodes.

## Introduction

With the rapid development of renewable energy of wind and solar power, the large-scale energy storage system has become more and more important (Manthiram et al., 2016; Chen et al., 2018; Wu et al., 2018; Wu C. et al., 2019; Xia et al., 2019). Among them, lithium ion batteries have been powering our daily life from mobile phones to electric vehicles (EVs), due to their high energy density, long cycle life and environmental friendly (Chen et al., 2014; Li et al., 2018; Wu L. et al., 2019; Ye et al., 2019). With the rapid development of high-performance silicon-carbon composite anodes (Su et al., 2018; Xiao et al., 2018), cathode materials become the technological bottleneck for obtaining high performance lithium-ion batteries (Myung et al., 2016; Chen Z. et al., 2017; Xu et al., 2017; Zheng et al., 2019). Among the various types of layered cathodes, LiNi_0.8_Co_0.1_Mn_0.1_O_2_ (NCM) is attracting an increasing amount of attention on account of its high specific capacity and low cost, compared to traditional LiCoO_2_ material (Zhang et al., 2012; Li et al., 2016a; Chen M. et al., 2017; Yang et al., 2019).

However, the long-term capacity retention of NCM is not satisfactory for large-scale application, which can be ascribed to the undesired side reactions between the highly reactive species Ni^4+^ and liquid electrolyte at the interface of the cathodes, triggering the irreversible phase transition from initial layered R-3m phase to spinel Fd-3m phase and further to the rock-salt phase (Nam et al., 2013; Lin et al., 2014a,b). Worse yet, the NiO passivation layer will increase the impedance, hindering the electrochemical kinetics, and resulting in the deterioration of electrochemical performance (Meng et al., 2017; Ryu et al., 2018a).

During the extraction of Lithium-ion, Ni-rich materials undergoes a series of phase transitions: The original layered structure (H1) transforms to the monoclinic phase (M), the second hexagonal phase (H2), and the third hexagonal phase (H3) (He et al., 2018; Gao et al., 2019; Wu et al., 2019a). It has been reported that the H2-H3 transition will cause detrimental lattice shrinkage along the c-direction, resulting in the volume change and the local stress accumulation, and further leading to the microcracks generation and propagation in secondary particles (Lee et al., 2014; Sun and Manthiram, 2017; Yoon et al., 2018). Ryu et al. (2018b) reported the cross-sectional SEM images of the charged LiNi_0.90_Co_0.05_Mn_0.05_O_2_ particles, numerous cracks emanating from the particle core and some cracks traverse across the entire particle and nearly fracture the secondary particle. The produced cracks create fresh surface where phase transitions and corrosion as well as side reactions occur, thereby further accelerating the structural degradation of the cathodes.

Such severe structural collapse affected by mechanical strain associated with the poor irreversibility of the H2-H3 phase transition during cycling. Cations substitutions have been regarded as promising way to overcome these challenges and enhance the structural stability of Ni-rich materials (Xia et al., 2018; Li et al., 2019; Susai et al., 2019; Weigel et al., 2019). Wu et al. (2019b) demonstrated that by doping Ti^4+^ in LiNi_0.9_Co_0.1_O_2_ materials, the improved reversibility of the H2-H3 phase transitions and the lossless H3 phase suppress the generation of microcracks and structural degradations. It is also reported that, by doping with boron, the LiNi_0.9_Co_0.05_Mn_0.05_O_2_ shows no visible cracks, which is consistent with its good reversibility of H2-H3, attesting to the beneficial effect of boron doping and enables deflection of the internal strain posed by the phase transition (Park et al., 2018).

In this work, the structural and electrochemical performances of Ti-doped LiNi_0.8_Co_0.1_Mn_0.1_O_2_ (0, 0.5, 1, 2%) have been systematically studied. The Ti doping can keep the layered structure materials with one phase. Among them, the Li(Ni_0.8_Co_0.1_Mn_0.1_)_0.99_Ti_0.01_O_2_ sample exhibits the best electrochemical performance. Within a voltage window of 2.7**–**4.3 V, the Li(Ni_0.8_Co_0.1_Mn_0.1_)_0.99_Ti_0.01_O_2_ sample maintains a reversible capacity of 151.01 mAh·g^−1^ with 83.98% capacity retention after 200 cycles, corresponding 0.08% decay per cycle. The improved electrochemical performance can be ascribed to the enhanced abilities of electronic transition and Li ion diffusion. More importantly, the reversibility of H2-H3 phase transitions and the lossless H3 phase during prolonged cycling can also improve the electrochemical performance of Ni-rich cathodes.

## Experimental Section

### Materials Synthesis

The pristine LiNi_0.8_Co_0.1_Mn_0.1_O_2_ (denoted as NCM) spherical material was synthesized by solid-state method. Stoichiometric ratio of LiOH·H_2_O and Ni_0.8_Co_0.1_Mn_0.1_(OH)_2_ precursor were mixed and ground in an agate mortar. Then, under oxygen atmosphere, the mixtures were precalcined at 480°C for 5 h and calcined at 830°C for 12 h with a heating rate of 5°C·min^−1^. Next, the sample was cooled to room temperature naturally in a tube furnace.

The 0.5, 1, 2 mol% Ti-doped NCM (denoted as Ti-0.5, Ti-1, Ti-2) samples were prepared by the following steps. Firstly, the stoichiometric amount of C_16_H_36_O_4_Ti was dissolved into 60 mL absolute ethanol, then stirring continuously in water bath at the temperature of 60°C. Secondly, the LiOH·H_2_O were added to the solution and kept stirring for about 30 min, subsequently the Ni_0.8_Co_0.1_Mn_0.1_(OH)_2_ precursor was added. Afterward, the mixed solution was dried overnight at 120°C. Finally, the Ti-doped samples were obtained by calcining under the same condition as NCM.

### Materials Characterization

The crystalline structures of all the samples were determined by X-ray diffraction (XRD, Bruker D8 advance) using Cu Kα radiation in the 2θ range of 10–90° with a scan rate of 5°·min^−1^ and operated at 40 kV and 40 mA. Scanning electron microscopy (SEM, Nova NanoSEM-230) was performed to observe the particle morphologies of samples. The microstructures of the NCM and Ti-1 samples were examined by high-resolution transmission electron microscopy (HRTEM, FEI Talso 200s).

### Electrochemical Measurements

The electrochemical properties were measured using a CR2025 coin-type half cell. The working electrodes were prepared by mixing the active materials, acetylene black, and polyvinylidenedifluoride (PVDF) with a weight ratio of 8:1:1 in N-methyl-2-pyrrolidone (NMP). The resulting slurry was cast uniformly onto aluminum foil, followed by drying at 120°C in a vacuum oven for 4 h and pouched. The typical mass loading of positive electrode was about 2.3 mg·cm^−2^ with an electrode diameter of 12 mm. 1 M LiPF_6_ dissolved in a solution of dimethyl carbonate (DMC), ethylene carbonate (EC), and ethyl methyl carbonate (EMC) (volume ratio = 1:1:1) was used as the electrolyte. All the cells were assembled in an Ar-filled glovebox where the moisture and oxygen content is under 0.1 ppm by using a lithium metal anode and separator (Celgard 2500 polyethylene). Then, the cells were silenced before electrochemically tests. Electrochemical tests were performed on NEWARE BTS7.6 battery test system between 2.7 and 4.3 V vs. Li/Li^+^ at room temperature (25°C). The cycling performance was initially charged and discharged at 0.1 C (18 mA·g^−1^) with 2 cycles, then charged and discharged at 1 C in the subsequent cycles. The cyclic voltammetry (CV) was measured at a scan rate of 0.1 mV·s^−1^ within 2.7–4.5 V through an electrochemical workstation (Solartron 1470E). Electrochemical impedance spectroscopy (EIS) tests were conducted in the frequency range of 10^−3^-10^5^ Hz.

## Results and Discussion

**Figure 1:**
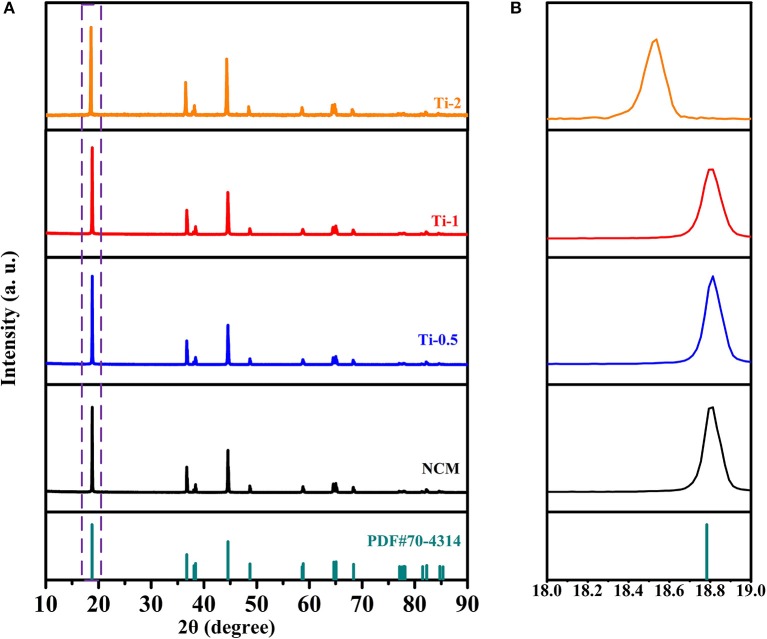
**(A)** XRD patterns of NCM, Ti-0.5, Ti-1, and Ti-2; **(B)** Corresponding enlarged patterns of (003) peak between 18 and 19°.

The structure characteristics of the NCM and Ti-doped NCM samples are analyzed by XRD measurements as shown in Figure 1A. All the diffraction peaks of all samples can be well indexed to the hexagonal α-NaFeO_2_ layered structure (PDF#70-4314) with R-3m space group, and no secondary phase is observed, indicating that Ti^4+^ is successfully incorporated into the bulk structure. Furthermore, the clear splitting of (006)/(102) and (108)/(110) couples for all samples manifest a well-developed layered structure. The partial magnified patterns in Figure 1B displays that the (003) diffraction peaks shift to a lower angle with increasing Ti-doped content, demonstrating lattice expansion caused by the successfully dopant of Ti^4+^. The result can also be proved by the increase of lattice parameter c and the cell volume as shown in Table 1.

**Table 1:** Lattice parameters of all samples obtained by XRD analysis.

**Samples**	**a (Å)**	**c (Å)**	**V (Å^**3**^)**
NCM	2.86390	14.16697	100.63
Ti-0.5	2.86714	14.16738	100.78
Ti-1	2.86805	14.16898	100.87
Ti-2	2.86914	14.17678	100.89

**Figure 2:**
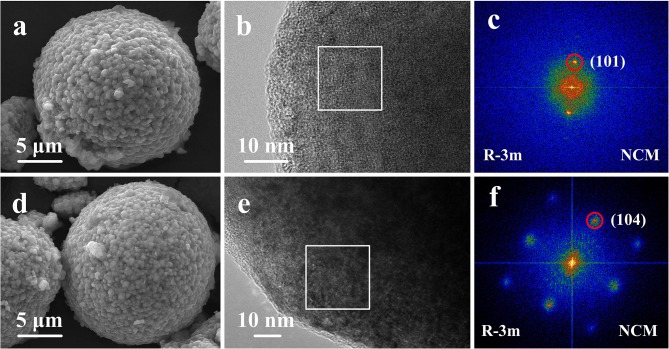
SEM images of the pristine NCM **(a)**, Ti-1 **(d)**; HRTEM images and Corresponding FFT images of the pristine NCM **(b,c)** and Ti-1 **(e,f)**.

The morphologies of the pristine NCM and Ti-1 samples are shown in Figures 2a,d. The pristine and modified samples images exhibit very similar morphology. It is found that the secondary particles are spherical with the size is ~12 μm in diameter, which are composed of densely packed primary particles. Apparently, the result indicates that Ti doping cannot change the particle morphology and particle size. To further investigate the effect of Ti doping on the microstructure of NCM sample, the HRTEM images of NCM (Figures 2b,c) and Ti-1 (Figures 2e,f) are performed. As shown, the rectangle region is selected as representative to observe the atomic-scale crystal structure, and the fast fourier transform (FFT) of the selected region are shown on the right. The FFT image of the NCM demonstrates that layered phase (space group R-3m) is successfully formed. For the Ti-1 sample, the diffraction spots of the corresponding FFT can be assigned to the (104) lattice plane, indicating that the microstructure cannot be changed by 1% Ti doping.

**Figure 3:**
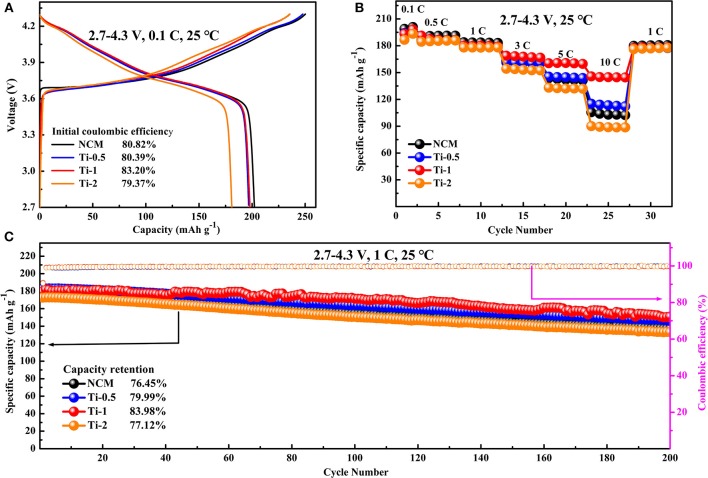
Electrochemical performance of all electrodes in the voltage range of 2.7–4.3 V at room temperature: **(A)** Initial charge-discharge profiles at 0.1 C. **(B)** Rate capabilities at various current densities. **(C)** Cycling performance at a current rate of 1 C.

Electrochemical properties of the pristine and Ti-doped NCM samples are tested in lithium-ion half-cells at room temperature. Figure 3A shows the initial charge/discharge profiles of all electrodes between 2.7 and 4.3 V at the rate of 0.1 C. It can be observed that all the discharge profiles are similar to each other. The initial discharge specific capacity is decreased slightly from 202.24 to 180.95 mAh·g^−1^ with the increasing of Ti amounts. However, the Ti-1 electrode shows the higher Coulombic efficiency of 83.20% than NCM electrode showing Coulombic efficiency of 80.82%, most probably owing to that suitable Ti doping can reduce the irreversible capacity.

The rate performances of the pristine and Ti-doped NCM electrodes at the current density ranging from 0.1 to 10 C are presented in Figure 3B. Comparing to the NCM electrode, the Ti-0.5 and Ti-1 electrodes show improved rate capability. Particularly, the Ti-1 electrode maintains a reversible capacity of 145.71 mAh·g^−1^ at high current rates of 10 C, while the NCM cathodes only deliver a specific capacity of 105.52 mAh·g^−1^. The remarkable rate property is mainly related to the slight enlargement along c axis by Ti doping, which benefits the fast Li ion diffusion. In addition, the cycling performances of all electrodes are compared at 1 C rate in the range of 2.7–4.3 V. As can be seen in Figure 3C, the NCM electrode shows fast capacity degradation from 179.03 to 136.88 mAh·g^−1^ after 200 cycles, corresponding to 76.45% capacity retention with 0.118% decay per cycle. In comparison, the Ti-doped electrodes possess the higher capacity retention than pristine electrode, specifically, the Ti-1 electrode maintains 151.01 mAh·g^−1^ with 83.98% capacity retention after 200 cycles, corresponding 0.08% decay per cycle.

**Figure 4:**
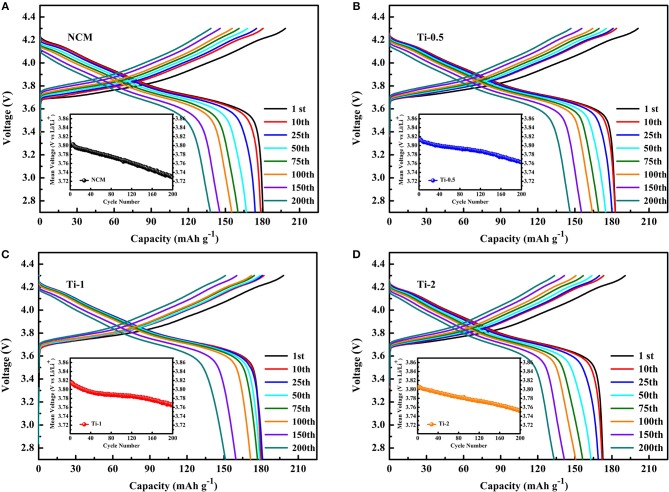
Charge-discharge curves of NCM **(A)**, Ti-0.5 **(B)**, Ti-1 **(C)**, and Ti-2 **(D)** at different cycle number (insets are the midpoint voltage vs. cycle numbers).

Figure 4 shows the charge-discharge curves of NCM, Ti-0.5, Ti-1, Ti-2 electrodes at different cycle number, respectively. It is distinctly observed that the reversible capacity and discharge voltage plateau of the NCM electrode declined rapidly with increasing cycle, while the Ti-1 electrode shows stable charge-discharge curves and discharge capacity, indicating that suitable Ti doping can effectively improve the cycle stability of NCM and alleviate the voltage reduction during cycling.

**Figure 5:**
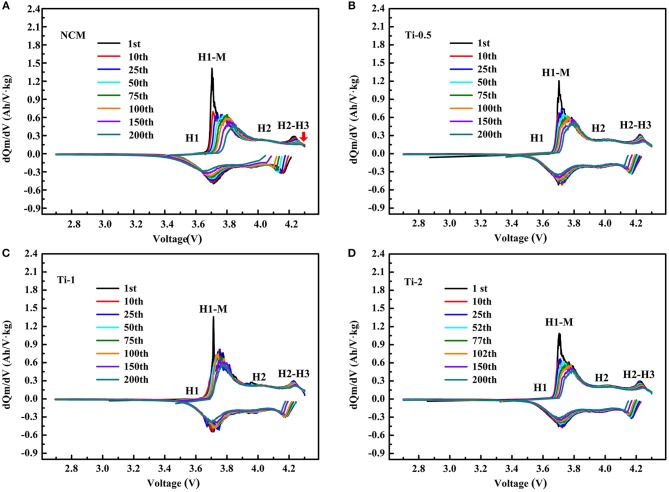
Differential capacity vs. cell potential curves of different numbers for the NCM **(A)**, Ti-0.5 **(B)**, Ti-1 **(C)**, and Ti-2 **(D)** electrodes.

To further understand the different charge-discharge behavior of the NCM and Ti-doped electrodes, the differential capacity (dQ/dV) curves are shown in Figure 5. All curves display three couples of redox peaks, which are ascribed to the phase transition of hexagonal (H1) to monoclinic (M), monoclinic (M) to hexagonal (H2), and hexagonal (H2) to hexagonal (H3) during the delithiation/lithiation processes. As shown in Figure 5A, the oxidation peaks for the pristine NCM shift to high potential significantly, while the reduction peaks shift to low potential during cycling, and the corresponding peaks areas are greatly decreased. As shows, the poor reversibility of H2-H3 phase transition and gradually loss of H3 phase associated with poor structural stability of NCM, should take responsibility for the faster capacity drop of the NCM sample. In contrast, the voltage hysteresis for the Ti-doped electrodes is substantially suppressed. For Ti-1, the negligible change of overlap of dQ/dV curves within 200 cycles suggests the lower polarization and better H2-H3 reversibility of the modified electrodes. In conclusion, the Ti-doped electrodes exhibit improved electrochemical properties, which can be attributed to the better structural stability due to the enhanced reversibility of H2-H3 phase transition.

**Figure 6:**
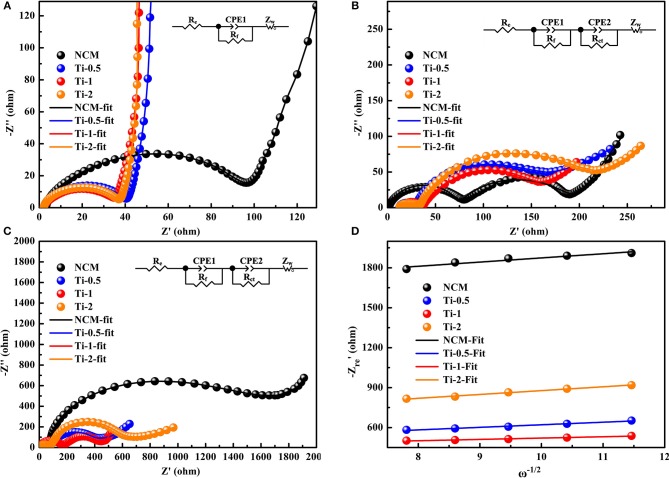
Nyquist plots of **(A)** as-prepared electrodes before cycling, **(B)** all electrodes at the charge state of 4.3V after 5th, **(C)** all electrodes at the charge state of 4.3V after 200th (insets are the equivalent circuit models), **(D)** the relationships between ${\text{Z}}^{\prime }{}_{\text{re}}$ and ω^−1/2^ based on the 200th cycles.

To understand the relationship between electrochemical degradation and resistance parameters, EIS measurements are conducted for all samples in a charged state of 4.3 V after different cycle numbers. Figures 6A–C show the Nyquist plots of the electrodes and corresponding equivalent circuit at the fresh cells, after 5 and 200 cycles, respectively. The curves consist of a semicircle and a sloped line in Figure 6A. The semicircle is regarded as the charge transfer resistance (R_ct_) and the followed sloped line relates to the Warburg impedance (Z_w_). Moreover, the intercept with real axis (Z′) is assigned to the solution resistance (R_s_). Differently, Figures 6B,C display an increased semicircle in the high-frequency region, which can be ascribed to the film resistance (R_f_) due to the solid electrolyte interface (SEI) film formed on the electrode during high-voltage cycling. The fitting results of the electrodes based on the equivalent circuit are summarized in Table 2. The R_ct_ of the NCM electrode in the fresh cell is 96.5 Ω, and the resistance increases dramatically after 200 cycles (1372 Ω). In comparison, the Ti-doped electrodes exhibit more stable values of R_ct_ during cycling, implying better structural stability. According to the relationships between ${\text{Z}}^{\prime }{}_{\text{re}}$ and ω^−1/2^ in the low-frequency region (Figure 6D), the lithium ion diffusion coefficient (D_Li+_) is calculated and the results are listed in Table 2 (Li et al., 2015, 2016b; Liu et al., 2019; Zhu et al., 2019). Notably, the Ti-1 electrode shows highest D_Li+_ values than that of the NCM electrode after 200 cycles, contributing to the best rate property among all electrodes. The EIS results demonstrate that the Ti-doped samples exhibit better kinetic behavior than those of the NCM, which can be related to the outstanding structural stability by the modification of Ti doping.

**Table 2:** The values of R_f_, R_ct_, and ${\text{D}}_{\text{Li}}^{+}$ for as-prepared electrodes and cycled electrode.

**Samples**	**As-prepared**	**5th**		**200th**		
	**R_**ct**_ (Ω)**	**R_**f**_ (Ω)**	**R_**ct**_ (Ω)**	**R_**f**_ (Ω)**	**R_**ct**_ (Ω)**	${\text{D}}_{\text{Li}}^{+}$ **(cm^**2**^ s^**−1**^)**
NCM	96.5	71.24	121.7	85	1372	7.13 × 10^−13^
Ti-0.5	39.3	31.3	102.1	87	351	7.79 × 10^−13^
Ti-1	37.2	32.4	103.2	155	263	2.01 × 10^−12^
Ti-2	35.3	31.7	163.2	124	445	7.19 × 10^−13^

**Figure 7:**
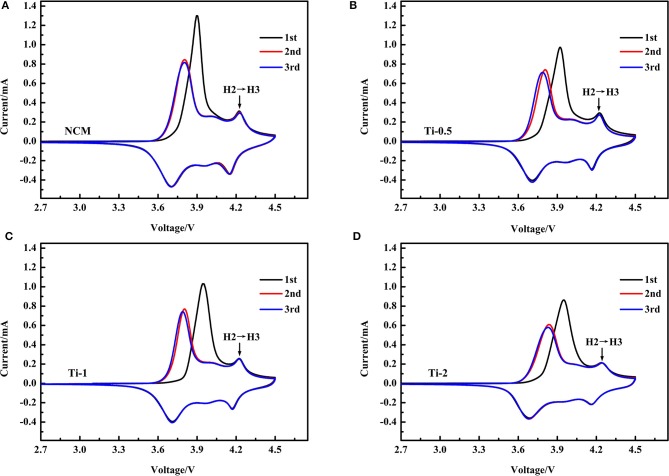
CV curves of NCM **(A)**, Ti-0.5 **(B)**, Ti-1 **(C)**, and Ti-2 **(D)** for first three cycles in the voltage range of 2.7–4.5 V.

To study the redox behavior associated with phase transition during battery operation, Cyclic voltammetry (CV) is employed at a scan rate of 0.1 mV·s^−1^ between 2.7 and 4.5 V. Figures 7A–D illustrate the first three CV curves of all samples, respectively. For these CV curves, three pairs of oxidation/reduction peaks are observed, which can be assigned to the phase transitions from H1 to M, H2 and H3. It is worth noting that the H2-H3 phase transition can induce sharp lattice contraction along c axis, result in the anisotropic changes of cell volume (Yang and Xia, 2016; Xu et al., 2019). Apparently, the peak intensity of the phase transition of H2-H3 gradually become weaker with increasing Ti concentration, implying that the Ti doping could suppress the detrimental phase transition during charging/discharging cycles. In other words, the structural stability of the NCM is improved by Ti doping.

## Conclusion

To address the problem related to the structural degradations of NCM, Ti is intentionally introduced to enhance the structural stability and electrochemical performance of LiNi_0.8_Co_0.1_Mn_0.1_O_2_. The Ti-doped LiNi_0.8_Co_0.1_Mn_0.1_O_2_ (0, 0.5, 1, 2%) composites are successfully synthesized via one-step calcination method. Among them, the Li(Ni_0.8_Co_0.1_Mn_0.1_)_0.99_Ti_0.01_O_2_ sample exhibits the best electrochemical performance, it maintains a reversible capacity of 151.01 mAh·g^−1^ after 200 cycles at 1 C with 83.98% capacity retention. The superior electrochemical performance can be ascribed to two aspects: (1) the enhanced reversibility of H2-H3 phase transitions and the lossless H3 phase during prolonged cycling; (2) the lower electrochemical impedance and the improved Li-ion diffusion ability.

## Data Availability

All datasets generated for this study are included in the manuscript/supplementary files.

## Author Contributions

LL, JieC, and HY: conceived the idea. JieC, HY, and LL: prepared all materials. JieC, HY, and TL: conducted SEM experiments. CL, JiaC, ZL, and LX: conducted XRD experiments. LL, JieC, and HY: analyzed the data. JieC and HY: wrote the manuscript. HT, ZC, and JD: commented on the manuscript. LL: supervised the implementation of the project.

### Conflict of Interest Statement

The authors declare that the research was conducted in the absence of any commercial or financial relationships that could be construed as a potential conflict of interest.
